# Optimising a MALDI-TOF MS database for the detection of xerophilic fungi across environments

**DOI:** 10.14324/111.444/ucloe.3244

**Published:** 2025-07-29

**Authors:** Christopher Campion, Victor Carp Kofoed, Jeppe Lund Nielsen, Anne Mette Madsen

**Affiliations:** 1 National Research Centre for the Working Environment, Copenhagen Ø, Denmark; 2 Department of Chemistry and Bioscience, Aalborg University, Denmark

**Keywords:** detection methods, cultural heritage, home exposure, indoor air, MALDI-TOF MS, museum, occupational exposure, Thermowood, xerophilic *Aspergillus* spp.

## Abstract

Xerophilic fungi can proliferate in dry conditions and have been detected in cultural heritage sites and libraries. To protect the staff from fungal exposure and ensure the preservation of heritage collections, research must be focused on improving detection protocols for xerophilic fungi. Matrix-assisted laser desorption/ionisation time-of-flight mass spectrometry provides a strong method for the identification of fungi; however, this is dependent on the reference database. The aim of this study was to investigate whether it is possible to develop a supplementary matrix-assisted laser desorption/ionisation time-of-flight mass spectrometry database of xerophilic/xerotolerant *Aspergillus* species. The database is intended to complement the current Bruker library; for this 19 *Aspergillus* species grown in four different broth media were included. The database was evaluated on samples from domestic homes, a museum and a warehouse. It was possible to create a database with mass spectra from the 19 species. For some species, it was possible to generate mass spectra from the four tested broth media, while other species required specific media and extended incubation time. Isolates from environmental samples identified by the Bruker fungi library were not misidentified by the supplementary database while some previously unidentified isolates (*Aspergillus conicus*, *Aspergillus domesticus*, *Aspergillus glabribes* and *Aspergillus pseudogracilis*) could be identified. Using low water-activity agar media had a profound effect on detection of these xerophilic/xerotolerant *Aspergillus* species. This work shows that it is possible to develop a supplementary matrix-assisted laser desorption/ionisation time-of-flight mass spectrometry database for the identification of xerophilic/xerotolerant *Aspergillus* species, and that low-water activity broth media are recommended for the construction of a database and the following application.

## Introduction

Uncontrolled fungal growth can compromise the preservation of heritage collections [1–4], leading to the deterioration of cultural heritage artefacts [5–12]. Furthermore, exposure to airborne fungi may pose a health risk for museum staff and visitors [13–15].

Common strategies to prevent fungal growth in museum artefacts, exhibition rooms and storage repositories include controlling indoor climate parameters such as temperature, humidity and sunlight, complemented by various methods to eliminate fungal growth [5,6]. However, xerophilic/xerotolerant species of the genus *Aspergillus* [16], *Cladosporium* [17] and *Penicillium* [18] have the ability to proliferate in extreme dry and water controlled [low water-activity (a_w_) or low relative humidity (RH)] conditions and many reports have identified xerophilic/xerotolerant fungi in museums, libraries, cultural heritage sites and their respective repositories [19–25] – some also being reported in places despite stringent climate-controlled environments [26]. These observations suggests that xerophilic/xerotolerant fungi growing at low RH/a_w_, might be overlooked when attempting to limit fungal growth in such environments.

Matrix-assisted laser desorption/ionisation-time-of-flight mass spectrometry (MALDI-TOF MS) provides an efficient method for the identification of cultivable fungal isolates. MALDI-TOF MS relies on measurements of the mass–charge ratio of extracted proteins as mass spectra. For microbial identification species-specific mass spectra of unknown isolates are compared to a reference spectra database. Depending on the degree of spectral similarity, this will result in identification to the genus or species level. The accuracy and specificity of MALDI-TOF MS identification therefore depend on the extent of the reference spectra database. The current Bruker database for MALDI-TOF MS contains several xerophilic/xerotolerant *Penicillium* and *Cladosporium* species, but only a few xerophilic/xerotolerant *Aspergillus* species. The Bruker database was initially made to support identification of hospital-related species. However, it is possible to add spectra of other species to a local library, thus a recent study has constructed a library on food-related species [27]. To better ensure a safe indoor air and work environment for the staff and ensure the preservation of our heritage collection, further research is needed to broaden a rapid detection and identification protocols of xerophilic/xerotolerant *Aspergillus* fungi.

This study aimed to evaluate whether it is possible to construct a supplementary MALDI-TOF MS database for the identification of xerophilic/xerotolerant *Aspergillus* spp. For this, we selected 19 xerophilic/xerotolerant *Aspergillus* spp., originating from the Institut for Bioteknologi (IBT) fungal culture collection (Table 1) and tested different broth media for cultivation. The supplementary database was evaluated on environmental samples, and different agar media were compared for the detection of xerophilic/xerotolerant species.

**Table 1.** Overview of xerophilic/xerotolerant fungi included in this study

Species*	Clade	IBT no.**	Additional reference no.	Country/source	MSPs	Spectra comprising MSP			
					Total	SAB***	CY20S	MY50	MY70
*A. destruens* ^a^	*A. conicus*	34818	NRRL 145T = CBS 593.91 = DTO 079-A8 = IMI 358691 = CCF 5462	USA, Maryland/maize seed	4	44	9	11	2
*A. villosus*	*A. conicus*	34822	NRRL 25813T = CCF 5531 = DTO 356-C9	UK, Kirkhill/unknown	4	40	6	9	4
*A. gracilis*	*A. conicus*	34817	NRRL 4962T = CBS 539. = DTO 351-H7 = CCF 5478 = ATCC 16906 = IMI 211393	USA, South Pacific/gun firing mechanism	4	21	5	4	1
*A. domesticus*	*A. conicus*	34814	DTO 079-F2T = CCF 5464 = NRRL 66616	Netherlands, Tiel/wallpaper	4	28	7	4	4
*A. conicus*	*A. conicus*	34288	EXF 7667	Slovenia, Ljubljana/Oil painting canvas	4	30	5	3	2
*A. pseudogracilis*	*A. conicus*	34813	CCF 5505T = EMSL No. 2765 = DTO 356-F3 = NRRL 66620	USA, California/child carrier	4	23	11	9	2
*A. reticulatus*	*A. penicillioides*	34819	CCF 5518 = EMSL No. 1272 = NRRL 58903	USA, Idaho/air	4	22	4	7	2
*A. canadensis*	*A. penicillioides*	34642	CCF 5548 = KAS 6194 = DTO 356-H9 = IBT 34520 = NRRL 66614	Canada, Wolfville/house dust	4	30	6	13	4
*A. clavatophorus*	*A. penicillioides*	34632	NRRL 25873 = CCF 5453	USA, Georgia/mouldy paper	1	32	-	-	-
*A. hordei*	*A. penicillioides*	34631	NRRL 25830 = CCF 5485	USA, Minnesota/insulation board	4	56	7	11	4
*A. infrequens*	*A. penicillioides*	34524	NRRL 25868T = CCF 5486 = DTO 356-D6	USA, Illinois/wheat	4	34	4	4	2
*A. penicillioides* ^b^	*A. penicillioides*	34815	CCF 5498 = EMSL No. 2440 = DTO 356-E7	USA, New Jersey/green fabric binders	3	21	-	5	4
*A. magnivesiculatus*	*A. penicillioides*	34816	NRRL 25866T = CCF 5488	Japan, Tokyo/fermented fish	4	52	12	14	12
*A. halophilicus*	*A. halophilicus*	34881	DTO 271-F4 = CCF 5825	Netherlands/textile	2	-	-	8	12
*A. vitricola*	*A. vitricola*	34272	EXF-10383 = CCF 5655	Slovenia, Ljubljana/oil painting canvas	4	11	5	4	2
*A. glabripes*	*A. vitricola*	34820	CCF 5474 = DTO 356-E8 = EMSL No. 2462 = NRRL 66618	Trinidad/office folder	4	18	5	5	6
*A. restrictus*	*A. restrictus*	33630	CBS 118.33	UK/cotton fabric	1	6	-	-	-
*A. caesiellus*	*A. restrictus*	34621	CCF 5448 = EMSL No. 1383	USA, Pennsylvania/air	4	14	4	1	2
*A. pachycaulis*	*A. restrictus*	34812	NRRL 25824 = CCF 5492 = DTO 356-D2 = IBT 34521 = IBT 34812	USA, Washington DC/unknown	4	17	4	5	3

The number of MSPs created for each fungal isolate and the number of spectra used to create the MSP for each of four broth media are presented.

*All species belong to the *Restrici* section.

**https://dtu.bio-aware.com.

***SAB broth.

^a^Updated name – *Aspergillus salinarum* (ex type of *Aspergillus destruens*, synonymy – *Phialosimplex salinarum* [28]).

^b^*A. penicillioides* is present in Bruker BDAL Filamentous Fungi Library version 4 (MaldiBiotyperDBUpdate_V4_Fungi-856(RUO)).

## Materials and methods

The study is divided into three parts. In the first part, a supplementary MALDI-TOF MS database was constructed using spectra generated from 19 xerophilic/xerotolerant *Aspergillus* spp. In the second part, previously stored spectra were analysed using the supplementary database to see whether previously identified or unidentified species were identified as some of the newly added species. In the third part, samples from different environments were cultivated and fungi were identified. The aim was to ensure that the supplementary database did not misidentify species which were already identified by the Bruker database, and to investigate the presence of xerophilic/xerotolerant species in these samples. Additionally, we aimed to evaluate the ability of different agar media to support the growth of xerophilic and xerotolerant fungi, from collected samples.

### Xerophilic/xerotolerant *Aspergillus* species for the supplementary database

A total of 19 xerophilic/xerotolerant identified *Aspergillus* species were obtained from the IBT fungal culture collection. These isolates were originally isolated in different countries and from different environments and materials such as air, house dust, mouldy paper, wallpaper and paintings (Table 1). These species were all the *Aspergillus* sect. *Restricti* and contained representatives of the *Aspergillus restrictus/Aspergillus conicus* clade, the *Aspergillus vitricola* clade, the *Aspergillus penicillioides* clade and the *Aspergillus halophilicus* clade [16].

The fungi were cultivated on Dichloran Glycerol Agar (DG18, Thermo Fisher Scientific Oxoid, Basingstoke, UK) [29] supplemented with 100 mg/L chloramphenicol. The species *A. halophilicus* was grown on Malt Yeast 50% Glucose Agar (MY50G) [30] as it does not grow well on DG18. All fungi were incubated at 25 °C for 14–21 days. Of the 19 *Aspergillus* spp. included in this study, *A. penicillioides* was already present in the Bruker BDAL Filamentous Fungi Library version 4 [MaldiBiotyperDBUpdate_V4_Fungi-856(RUO)] (Bruker, Bremen, Gemany), and a strain of *A. penicillioides* was included in the supplementary library as a positive control.

### Samples for MALDI-TOF MS

Protein extraction was performed using a modified version of the manufacture’s ethanol-formic acid extraction protocol. In brief, the fungal isolates were inoculated in 1.5 mL Eppendorf tubes containing a broth and incubated at room temperature for 1–14 days until visible growth was observed. Samples were inactivated by centrifugation, discarding the supernatant and resuspending the pellet in 70% ethanol. The ethanol was removed, and the sample was dried at room temperature, after which 5–20 μL (depending on pellet size) 70% formic acid was added. After 2 min, the same volume (5–20 μL, depending on pellet size) of acetonitrile was added. The suspension were then centrifuged and 1 μL of the supernatant was spotted onto a MALDI 96 main spectral profile (MSP) polished steel target plate (Bruker, Bremen, Germany) along with 1 μL (α-cyano-4-hydroxycinnamic acid (HCCA) matrix solution (#8255344, Merck, Darmstadt, Germany).

Spectra were acquired on a Microflex LT mass spectrometer (Bruker Daltonics Inc.), using the Bruker Biotyper software (v. 3.1) with the Filamentous Fungi Library version 4, MaldiBiotyperDBUpdate_V4_Fungi-856(RUO).

### MSP creation for the supplementary MALDI-TOF MS database

Each species in the supplementary database was represented by a series of MSPs, constructed by combining raw mass spectra obtained from the species isolates inoculated in four different liquid broth media [Sabouraud (SAB) agar [31], filtered MY50 [32], Czapek yeast 20% sucrose broth (CY20) [31] or filtered Malt Yeast 70% Glucose Broth (MY70)], at room temperature until growth was visible (which was for 3–5 days for all species except for *A. halophilicus*, which had to be incubated for 14 days, and *A. halophilicus* should have a double layer of matrix in contrast to other species).

For fungal identification, the standard procedure in this laboratory was to inoculate isolates in SAB broth, therefore, the majority of the MSPs were created based on raw mass spectra obtained from isolates inoculated in SAB broth. Our previous studies and other studies show that SAB supports growth of many different *Aspergillus* species [31,33]. MSPs were later created based on mass spectra obtained from isolates inoculated in MY50, MY70 or CY20 broth. Protein extraction was as described above.

Prior to MSP creation, all the raw spectra were visually examined using the flexAnalysis software (Bruker Daltonics Inc.), where spectra with outlier peaks and low mass to charge ratio (m/z) were excluded. Then, using the MALDI Biotyper Compass Explorer module, raw mass spectra were ‘preprocessed’ by default settings, which includes Mass Adjustment, Smoothing, Baseline Subtraction, Normalisation and Peak picking. This was followed by creating a principal component analysis plot of the spectra, enabling further examination and removal of outlying spectra. Finally, the MSPs obtained for all species were presented as an MSP dendrogram using the MALDI Biotyper Compass Explorer module (Bruker, Bremen, Germany). This analysis enables visualisation of how close the MSPs representing the different species are related to one another, reflected by an arbitrary distance level, normalised to a maximum of 1000. In summary, for each species, up to four reference MSPs were created based on the broth medium used for inoculation (SAB, MY50, CY20 and MY70).

### Search for xerophilic/xerotolerant *Aspergillus* species retrospectively

Previously obtained and stored mass spectra, originating from an unrelated study on drilling waste treatment plants, were used to perform a test of the supplementary database, using the Bruker Biotyper 3 software and the BDAL Filamentous Fungi Library version 4 [MaldiBiotyperDBUpdate_V4_Fungi-856(RUO)]. Exposure samples had previously been plated on DG18 and SAB agar. The number of mass spectra obtained from isolates grown on DG18 agar (n_DG18_) and SAB agar (n_SAB_) was 197 and 116, respectively. Further information on the study has been published elsewhere [34].

### Evaluation of the supplementary MALDI-TOF MS database on various environmental samples

The supplementary database was evaluated on environmental samples from three different environments, utilising different sampling techniques. The environments included in this study were domestic homes (n_samples_ = 27), a museum (n_samples_ = 15) and a warehouse (n_samples_ = 9). Environmental samples can be collected using different methods, depending on the environment and the aim of the research. Therefore, for each environment, a different sampling method was utilised. Air samples were taken in the warehouse using Gesamtstaubprobenahme (GSP, CIS by BGI Inc.) sampling actively at 3.5 L/min. Electrostatic dust collectors (EDC) were used in homes as passive samplers. In an open-air museum environmental surface swabs (eSwab collection and transport system designed for microbiological sampling) were taken from the surfaces of artefacts.

The warehouse was used for storage of gardening and roadwork equipment, as well as tools and machinery used by workers for the maintenance of public spaces. The warehouse was selected for this study, because fungi seemed to grow on the walls, and it is important to protect the workers from exposure. The warehouse is made of heat-treated pine wood (Thermowood), and fungal growth was suggested to be caused by fluctuations in the humidity. Personal GSPs (n_samples_ = 6) were carried by workers when they were present in the warehouse in the morning and sampled for 11–21 min and the stationary GSPs (n_samples_ = 3) sampled for 240 min at specified locations in the warehouse. The GSP samplers were mounted with 37 mm polycarbonate filters (pore size of 0.8/1 μm; Merck, Damstadt Germany).

The museum is an open-air facility consisting of historical houses, buildings and indoor exhibitions representing various eras throughout history. Here, surface samples were collected using eSwab (Copan’s Liquid Amies Elution Swab, eSwab; Copan, Brescia, Italy) from artefacts in various locations. The museum was selected because the staff expressed concerns that the furniture and artefacts were colonised by fungi. Samples were collected from surfaces that appeared to be colonised by fungi, surfaces that appeared clean and surfaces covered in dust.

The homes were randomly selected and with no described water damage. The home environments utilised long-term passive sampling (11–19 days) of dust using an EDC cloth (ZEEMAN, Alphen, The Netherlands, surface exposure area: 209 m^2^).

### Quantification and identification of fungi from environmental samples

Sampled material was extracted using an extraction solution [MilliQ water, 0.85% sodium chloride (NaCl) and 0.05% Tween80] while shaking (GSP and eSwab – 15 min at 500 rpm; EDC – 60 min at 300 rpm). All samples were stored at −80 °C in 30% glycerol until plated.

The GSP samples were plated on DG18 and MY50 agar. The eSwab samples were plated on SAB, DG18 and MY50 agar. The EDC samples were plated on DG18, MY50 and malt extract agar (MEA). In the case of agar plates being overgrown, making colony counting and isolation impossible, the samples were plated in 10 × dilutions (MilliQ water, 0.85% NaCl, 0.001% Bac. peptone, Merck KGaA, Darmstadt, Germany). All agar plates were incubated at 25°C; DG18, SAB, MEA for 1 week and MY50 for 3 weeks.

Following incubation, fungal colonies were counted. Fungal concentrations from EDC and GSP samples were calculated, taking into account the number of colonies, how much samples were diluted, sampling time and volume extracted, and for GSP samples also the flow rate.

The fungal colonies were prepared for MALDI-TOF MS as described above and identified using the Bruker database as well as the extended database. Identification of isolates were analysed as technical duplicates, with the cut-offs: identification (ID) scores <1.75 were unidentified, ID scores 1.75–1.85 were identified at genus level and ID scores >1.58 identified to species level.

### Data analysis and visualisation

Data analysis was conducted using R v.4.2.3 [35] using the packages ‘tidyverse’ [36], ampvis2 [37] and ggplot2 [38] for data handling and visualisation. Data on fungal species (xerophilic/xerotolerant out of total) on different agar media were compared using Fisher’s Exact Test.

## Results

### Construction of a supplementary MALDI-TOF MS database

MSPs were successfully created from all four broth media for 15 out of the 19 xerophilic/xerotolerant *Aspergillus* species. However, for *Aspergillus clavatophorus*, *A. restrictus*, *A. penicillioides* and *A. halophilicus*, it was not possible to obtain spectra from all media (Table 1). It was only possible to obtain spectra of *A. halophilicus* if the fungus was inoculated in broth media with low water activity (MY50 and MY70) for 10 to 14 days. In contrast, spectra of *A. restrictus*, *A. clavatophorus* and *A. penicillioides* were of insufficient quality when inoculated in MY50, MY70 or CY20 (Table 1).

**Figure 1:**
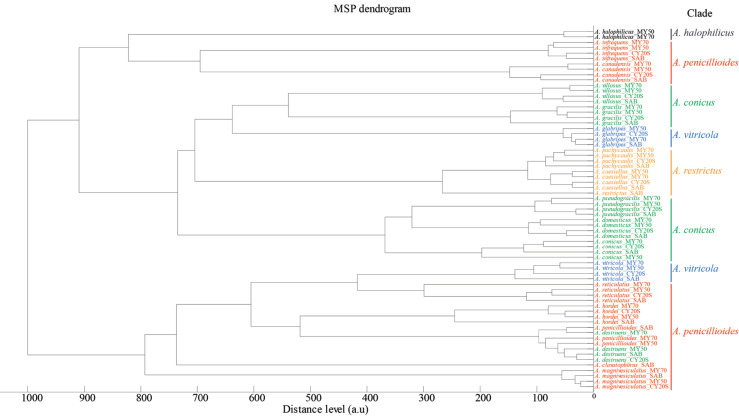
AN MSP dendrogram based on MSPs of the 19 species of xerophilic/xerotolerant *Aspergillus*, using the MALDI Biotyper Compass Explorer module. The distance level [arbitrary unit (a.u.), normalised to a max of 1000], reflects the differences between species and how related the MSPs are to one another. Clade designation based on genomic data is indicated by clade-specific colours and marked on the right-hand side of the image [16].

Discrimination between the 19 xerophilic/xerotolerant *Aspergillus* species of the supplementary database was evaluated by an MSP dendrogram (Fig. 1). The majority of the MSPs formed species-specific groups, with the exception of *Aspergillus destruens* and *Aspergillus penicilloides*, which were indistinguishable from each other. Generally, the MSP species groups formed clades, with similar patterns to previously published data based on genomic data, albeit with some discrepancies [16]. The *Aspergillus restrictus* clade, consisting of *Aspergillus pachycaulis*, *Aspergillus caesiellus* and *A. restrictus* were grouped together, forming a distinct cluster. The *A. conicus* clade (*A. villosus*, *Aspergillus gracilis*, *Aspergillus domesticus*, *Aspergillus pseudogracilis*, *A. conicus*, *A. destruens*), instead of forming one clade, formed two clades of *A. villosus*, *A. gracilis* and *A. domesticus*, *A. conicus*, *A. pseudogracilis*, with *A. destruens* being indistinguishable from *A. penicillioides* from the *A. penicillioides* clade. The two species included from the *A. vitricola* clade (*Aspergillus glabripes*, *A. vitricola*) were grouped with species of the *A. penicillioides* clade and *A. conicus* clade. The species included from the *A. penicillioides* clade (*Aspergillus magnivesiculatus*, *Aspergillus hordei*, *A. penicillioides*, *A. clavatophorus*, *Aspergillus infrequens*, *A. canadensis*), were split into two distinct clades.

When queried against the Bruker fungi database, only *A. penicillioides*, which already was present in library, was correctly identified. The remaining MSPs were not identified, as expected. The MSPs of *A. destruens* were indistinguishable from *A. penicillioides* and would be identified as *A. penicillioides* by the Bruker fungi library. The MSPs were kept in the supplementary database regardless.

### Search for xerophilic/xerotolerant *Aspergillus* species retrospectively in stored mass spectra

**Figure A1:**
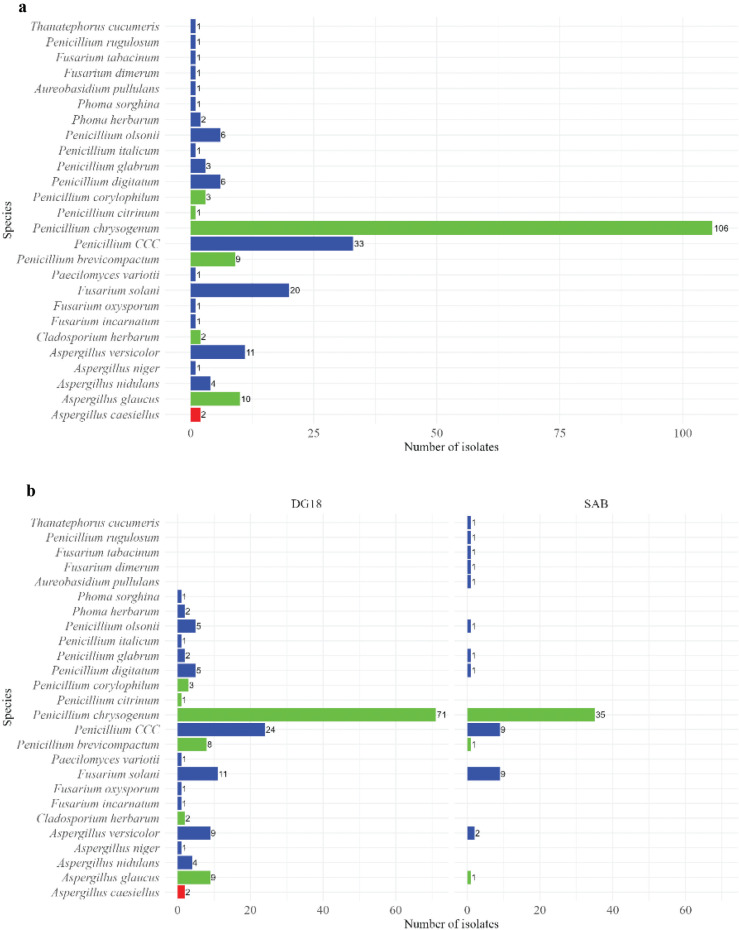
Retrospective search in previous obtained mass spectra for xerophilic/xerotolerant fungi. The mass spectra are from fungi in drilling waste plants. Fungi from the supplementary database are highlighted in red, xerophilic/xerotolerant species present in the Bruker library in green, non-xerophilic/xerotolerant fungi in blue. (a) Total species identified. (b) Species identified based on agar media (n_DG18_ = 197; n_SAB_ = 116). *Penicillium CCC* is an abbreviation for *P. camemberti*, *P. commune* or *P. cyclopium*, which cannot be distinguished by the MALDI-TOF MS library.

The supplementary database was used to identify mass spectra previously obtained from a study on drilling waste treatment plants [34]. Xerophilic/xerotolerant fungi had been reported in this environment, therefore, a total of 313 mass spectra originating from isolates grown on DG18 and/or SAB agar plates (n_DG18_ = 197; n_SAB_ = 116), respectively, were attempted to be re-identified, using the supplementary database. Of these, two previously unidentified isolates (originating from the same DG18 agar plate) were now identified as *A. caesiellus*, with ID scores >2.00 (Fig. A1).

The two agar media used in this study appeared to affect the total number of xerophilic/xerotolerant species detected (Fig. A1). We observed that the samples inoculated on DG18 agar plates, contained a higher fraction of xerophilic/xerotolerant species (n = 7/21) of the genera *Penicillium*, *Cladosporium* and *Aspergillus* when compared to the samples detected on SAB agar plates (n = 3/14) – although the differences were not significant (*p* = 0.70).

### Xerophilic *Aspergillus* species in environmental samples

**Figure 2:**
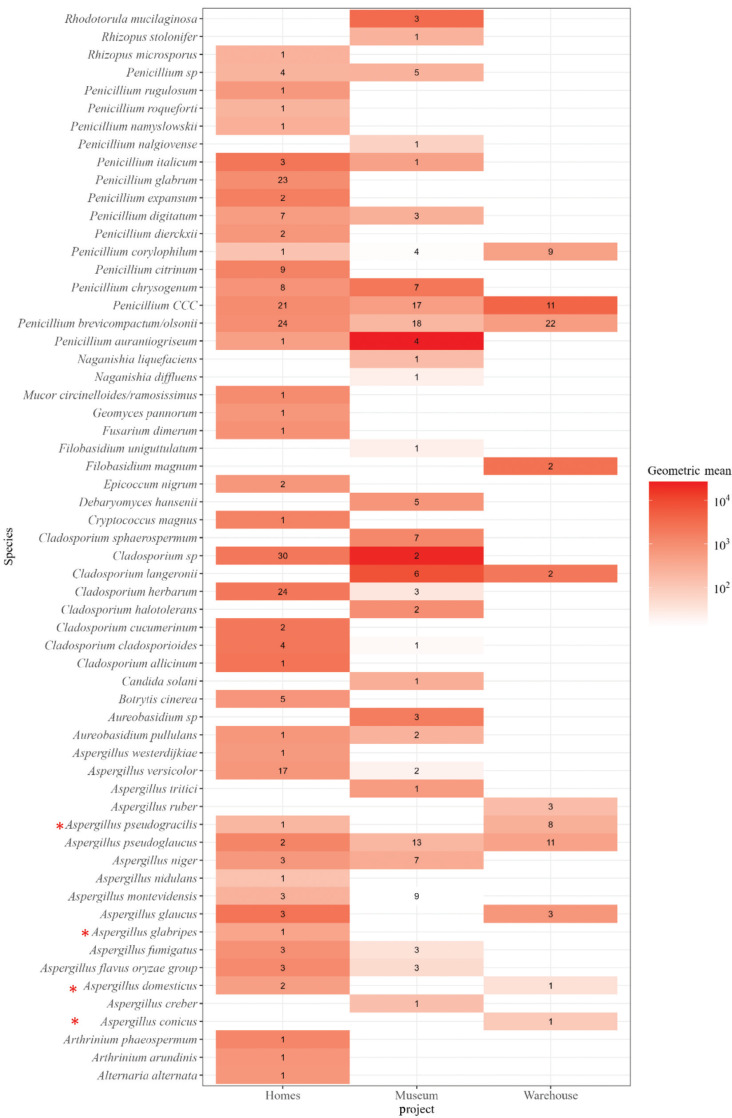
Heatmap of fungal species identified (y-axis) across three environments (x-axis). Numbers represent the number of isolates found. The colour gradient represents the log10-transformed geometric mean (GM) concentration (CFU/m^2^/day, CFU/location and CFU/m^3^). *Penicillium CCC* is an abbreviation for *P. camemberti*, *P. commune* or *P. cyclopium*, which cannot be distinguished by MALDI-TOF MS. Species marked with * are identified by the supplementary database.

The supplementary database was further evaluated using environmental samples collected from domestic homes (n = 27, via EDC), a museum (n = 15, via eSwab) and a warehouse (n = 9, via GSP) (Fig. 2). Across all environments, isolates identified to species level by the Bruker fungi library were not misidentified by the new supplementary database.

**Figure A2:**
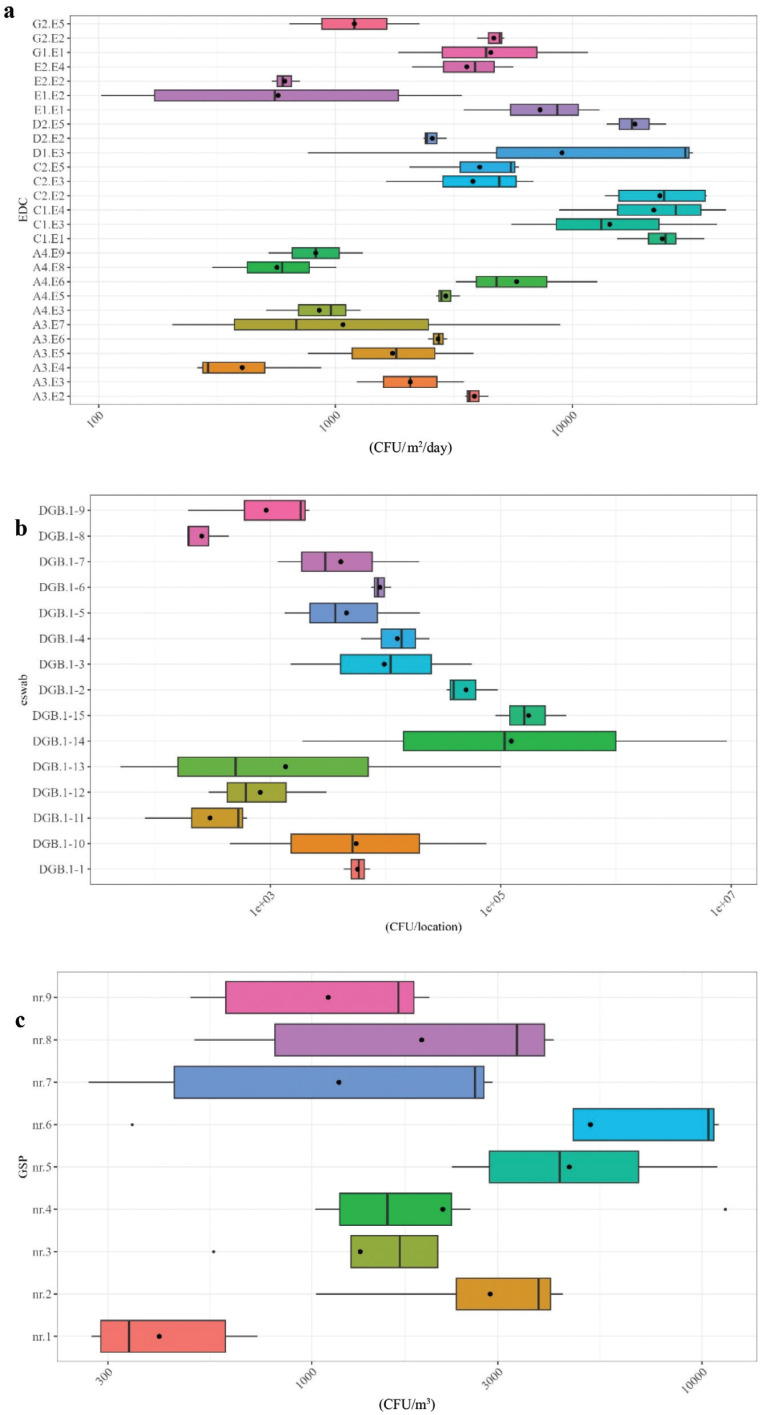
Concentrations of fungi across three environments, each using different sampling techniques: (a) EDC, homes; (b) surface eSwab, museum; C GSP air samplers, warehouse. The x-axis shows log10-transformed concentrations (CFU/m^2^/day, CFU/location and CFU/m^3^). The black dots represent GM values of the sample, while the smaller dots represent outliers.

The fungal concentration of the air exposure samplers (GSP) was between 268 and 1.15 × 10^4^ colony forming units (CFU)/m^3^. The fungal concentration of the sedimented dust (EDC) was between 205 and 4.45 × 10^4^ CFU/m^2^/day. The fungal surface concentrations (eSwab) were between 50 and 9.2 × 10^6^ CFU/location range (Fig. A2).

**Figure 3:**
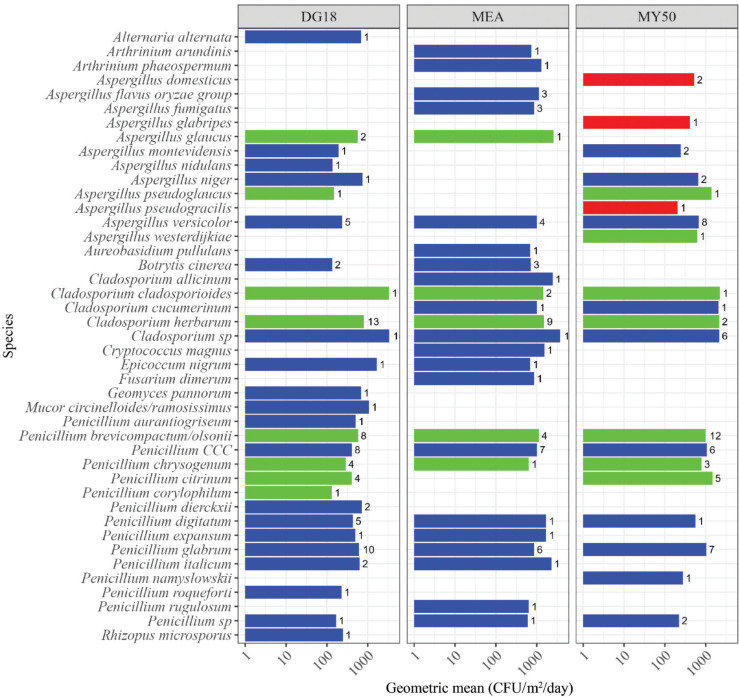
Fungal species in homes (EDC samples) on DG18, MEA and MY50 agar. Numbers represent the number of isolates in samples. The concentration is represented as GM of samples. Xerophilic/xerotolerant fungi from the supplementary database are highlighted in red, xerophilic/xerotolerant species present in the Bruker fungi library in green and other fungal species in blue. *Penicillium CCC* is an abbreviation for *P. camemberti*, *P. commune* or *P. cyclopium*, which cannot be distinguished by MALDI-TOF MS.

**Figure 4:**
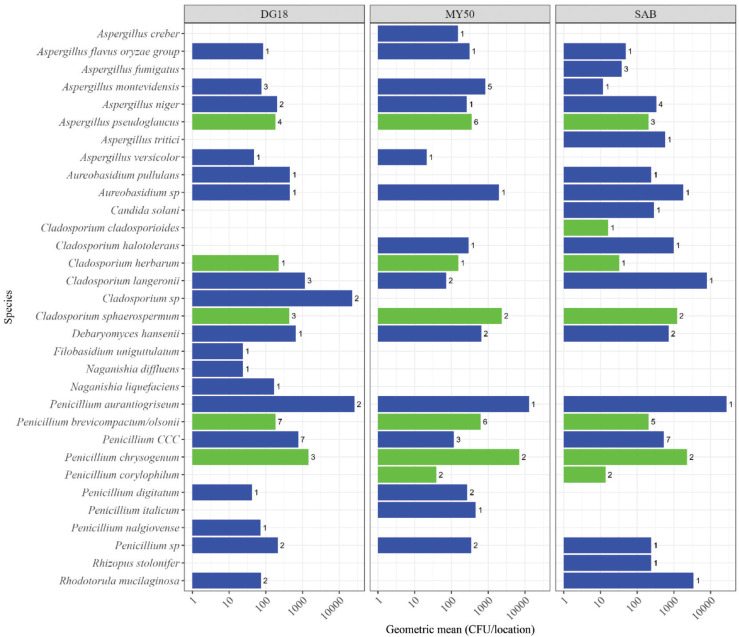
Fungal species in surface eSwab from a museum on DG18, MY50 and SAB agar. Numbers represent the no. of isolates in samples. The concentration is represented as GM of samples. Xerophilic/xerotolerant species present in the Bruker fungi library are highlighted in green, and other fungal species in blue. *Penicillium CCC* is an abbreviation for *P. camemberti*, *P. commune* or *P. cyclopium*, which cannot be distinguished by MALDI-TOF MS.

**Figure 5:**
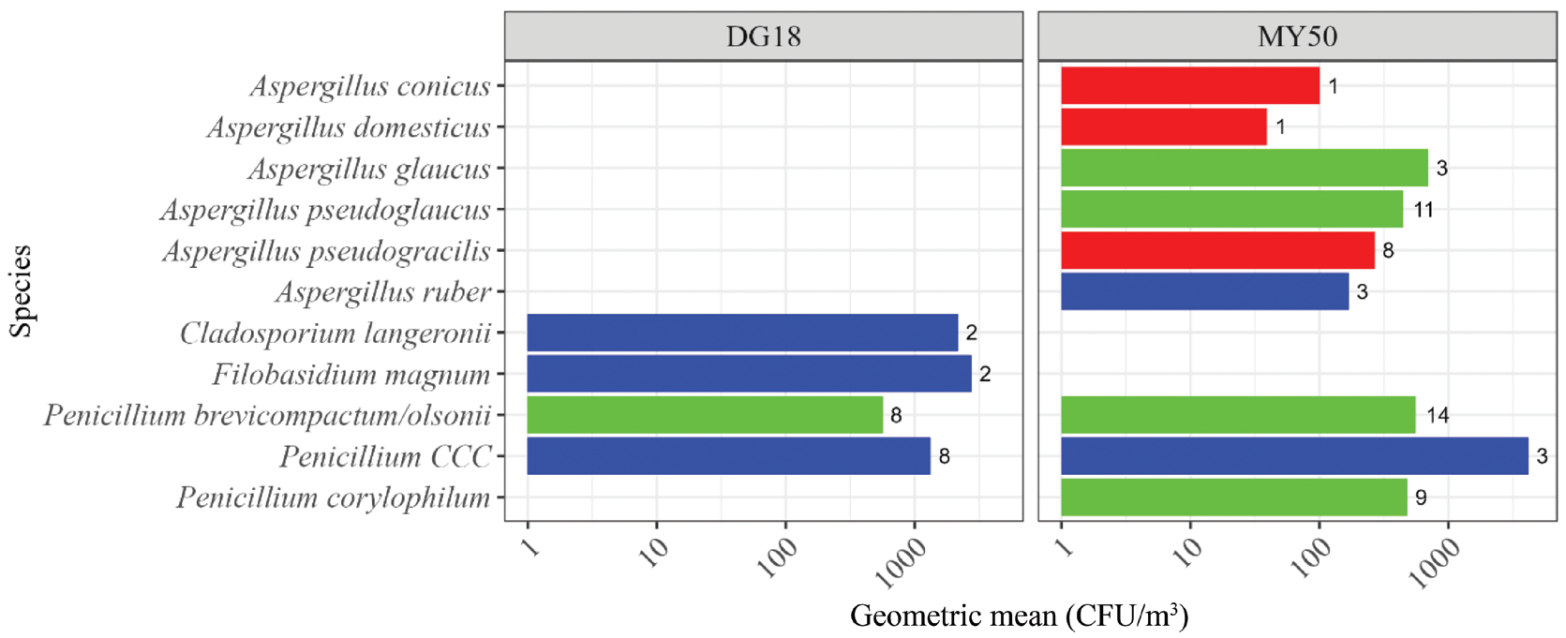
Fungal species in personal GSP samples from a warehouse on DG18 and MY50 agar media. Numbers represent the no. of isolates in samples. The concentration is represented as GM of samples. Xerophilic/xerotolerant fungi from the supplementary database are highlighted in red, xerophilic/xerotolerant species present in the Bruker fungi library in green, and other fungal species in blue. *Penicillium CCC* is an abbreviation for *P. camemberti*, *P. commune* or *P. cyclopium*, which cannot be distinguished by MALDI-TOF MS.

Xerophilic *Aspergillus* species, not present in the Bruker library, were detected in the home and the warehouse samples when using the supplementary database. The xerophilic *Aspergillus* species identified using the supplementary database were *A. pseudogracilis* (nine isolates), *A. glabripes* (one isolate), *A. domesticus* (three isolates) and *A. conicus* (one isolate). *Aspergillus domesticus* and *A. pseudogracilis* were identified in two of the environments tested (Figs 3–5). Other xerophilic/xerotolerant fungi were identified from these environments using the Bruker library, including *A. glaucus* (six isolates), *Penicillium brevicompactum/olsonii* (64 isolates), *Penicillium chrysogenum* (15 isolates), *Penicillium corylophilium* (14 isolates) and *Cladosporium herbarum* (27 isolates) identified in several samples (Figs 3–5).

The home environments were dominated by the genus *Cladosporium*, with *Cladosporium cladosporioides* and *C. herbarum* being present in high concentrations of 7.0 × 10^3^ CFU/m^2^/day and 4.4 × 10^3^ CFU/m^2^/day, respectively. Species of *Penicillium* were also present in high concentrations. The three xerophilic *Aspergillus* species identified from these environments, *A. domesticus*, *A. glabripes* and *A. pseudogracilis*, using the supplementary database were reported in concentrations of 520 CFU/day, 410 CFU/day and 210 CFU/day, respectively (Fig. 3).

The most abundant fungi detected in the museum were *Penicillium aurantiogriseum* (6.5 × 10^4^ CFU/location), *P. chrysogenum* (1.1 × 10^4^ CFU/location) and isolates belonging to the *Cladosporium* genus (2.2 × 10^4^ CFU/location). No additional xerophilic *Aspergillus* species were identified from the museum samples using the supplementary database (Fig. 4).

The warehouse samples were dominated by *Penicillium camemberti commune cyclopium* (*Penicillium CCC*) (5.5 × 10^3^ CFU/m^3^), *Cladosporium langeronii* (2.2 × 10^3^ CFU/m^3^) and the yeast *Filibasidium magnum* (2.8 × 10^3^ CFU/m^3^). In this environment, *A. pseudogracilis*, *A. domesticus* and *A. conicus* were found in concentrations of 270 CFU/m^3^, 39 CFU/m^3^ and 100 CFU/m^3^, respectively (Fig. 5).

### Effect of media on detection of xerophilic *Aspergillus* species in environmental samples

The various agar media used for fungal growth tended to affect the detection of xerophilic fungi (Figs 3–5). In the home environment, a larger fraction of the detected fungi tended to be xerophilic species when using MY50 (10/21 = 48% of species) and DG18 (8/29 = 27% of species), contra MEA (5/26 = 19% of species); (for MY50 vs MEA, *p* = 0.059). None of the xerophilic *Aspergillus* species included in the supplementary database were detected on DG18 or MEA. A similar trend was observed in the warehouse environment with 7/10 (70%) of species on MY50 being xerophilic species compared to 1/5 (20%) of species on DG18 (*p* = 0.12). This trend, however, was not observed in the museum environment (MY50, 6/22 = 27% of species; DG18, 5/25 = 20% of species; SAB, 7/25 = 28% of species) (*p* = 0.77).

## Discussion

In this study, a MALDI-TOF MS supplementary database consisting of 19 xerophilic *Aspergillus* species was constructed for the purpose of complementing the current Bruker fungi library for identification of these species in environmental samples.

### Construction of a supplementary MALDI-TOF MS database of selected xerophilic *Aspergillus* species

Culture condition and sample preparation methods can influence the mass spectrum obtained [39,40]. Therefore, the database was constructed using MSPs based on raw mass spectra originating from isolates inoculated in four different broth media. Raw mass spectra of the highly xerophilic fungus, *A. halophilicus*, were only obtained when the fungus was inoculated in broth media of low water activity for at least 14 days. The inability to obtain raw mass spectra from *A. halophilicus* might be partly explained by it being obligately xerophilic and not readily cultured in high water-activity media [41–44]. This suggests that, when working with environments where extreme/obligate xerophilic fungi thrive, it is relevant to re-evaluate the protocols (i.e., inoculation media and incubation time) used to inoculate unknown fungal isolates, to obtain mass spectra and hence ensure species identification.

Importantly, none of the MSPs of the supplementary database matched entries in the Bruker fungi library except for the species *A. destruens* identified as *A. penicillioides.* The MSP dendrogram showed that the MSPs formed species-specific clusters of the various xerophilic *Aspergillus* species. An exception of the species-specific clusters was *A. penicillioides* and *A. destruens*, for which MSPs were indistinguishable. By the time of submission, we observed that the species designation was updated for *A. destruens* (strain IBT no. 34818), which is now designated *Aspergillus salinarum*. This species is a halophilic fungus isolated from a hypersaline environment (Table 1). However, this does not explain why MSPs of these two species are indistinguishable.

Regarding the overall clade structure of the MSP dendrogram, some disagreements in the phylogeny between proteomic-based data and published phylogenetic data based on genomic data were observed [16]. It should be noted that the supplementary database was based on a single strain per fungal species, which is insufficient for taxonomic characterisation of fungal species. Future studies, improving the supplementary database, by including biological replicates, will be relevant to provide better characterisation and clarify discrepancies.

### Testing the supplementary MALDI-TOF MS database

The supplementary database was first tested on previously acquired spectra from a study on drilling waste treatment facilities. Two unidentified isolates were now identified as *A. caesiellus*, demonstrating how already performed studies can easily be revised with supplementary fungal databases. Isolates identified to species level by the Bruker fungi library were not identified by the supplementary database, and thus it did not lead to misidentification.

### Testing the supplementary MALDI-TOF MS database on environmental samples

The supplementary database was used to complement the Bruker database from three different environments. Isolates of xerophilic *Aspergillus* species were identified in two of these environments (homes and warehouse).

The museum samples did not reveal any xerophilic *Aspergillus* species using the supplementary database, despite other xerophilic/xerotolerant species being identified by the Bruker database from this environment. It should be mentioned that the museum environment from which these samples originate was not a closed, confined or climate-controlled site, and may not have favoured the growth of the specific xerophilic *Aspergillus* species included in this study.

The warehouse was selected for this study because the walls were described as being dry and yet had fungal growth on the walls, and therefore the presence of airborne xerophilic fungi could be expected to be present. In line with this, the supplementary database detected two species, *A. glabripes* and *A. pseudogracilis*. Other xerophilic/xerotolerant were also identified from this environment by the Bruker database, including *A. glaucus*, *Aspergillus pseudoglaucus*, *P. brevicompactum* and *Penicillium corylophilum*. The low number of fungal species detected in this environment could be explained by the relatively short sampling time for the GSP samplers [11–21 min for the personal samplers (n = 6) and 240 min for the stationary samplers (n = 3)], resulting in a small sampled volume. The sampling for personal samplers covered the period the workers had tasks in the warehouse in the morning.

We analysed samples from 27 randomly selected homes. The xerophilic *Aspergillus* species identified by the supplementary database (*A. domesticus*, *A. glabripes* and *A. pseudogracilis*) constituted only a small part of the potential exposure to fungi in the homes. This is in accordance with what could be expected, as fungi in home environments may have several sources and not only sources supporting growth of xerophilic fungi. The relatively low concentration of xerophilic *Aspergillus* species, on the one hand, suggests that these species do not pose a problem in these homes. On the other hand, their presence, combined with the fact that methods selecting for these xerophilic fungi have not been widely used in previous studies in homes, highlights the need for further studies on their prevalence in homes. Previous studies in homes also using EDCs for sampling have typically used DG18 agar [45] and SAB agar [46]. The most common species observed are in accordance with what has previously been found in homes, such as certain *Cladosporium* (*Cladosporium sphaerospermum*, *C. herbarum*, *Cladosporium cladosporides)*, *Penicillium* (*P. brevicompactum*, *P. camemberti*, *P. chrysogenum*, *Penicillium citrinum*, *Penicillium commume*, *Penicillium glabrum*, *P. olsonii*) and *Aspergillus* (*A. glaucus*) species [17,47,48].

### Effect of media on isolation and identification of xerophilic fungi

The type of agar media tended to have an effect on detection of xerophilic/xerotolerant species. With the exception of *A. caesiellus* (Fig. A1), all xerophilic *Aspergillus* species from the supplementary database were only detected on MY50. Some xerophilic/tolerant species, such as *P. brevicompactum/olsonii*, *P. chrysogenum*, *C. cladosporioides*, *C. herbarum*, *A. pseudoglaucus* and *A. glaucus*, were detected regardless of the media used. It should be noted that the MY50 plates were incubated longer than DG18, MEA and SAB agar plates as it takes more time for fungi to grow on this water-restricted medium. One explanation as to why the xerophilic *Aspergillus* species in the supplementary database were not detected on DG18 (with exception of *A. caesiellus*, Fig. A1), and that fewer xerophilic/xerotolerant fungi were observed on other media, might be that they were outcompeted by faster growing species on less restrictive media.

As described previously in this discussion, the broth medium also has an impact on the creation of MSPs for the new database, and raw mass spectra of *A. halophilicus* could only be obtained if it was inoculated in MY50 or MY70 while *A. clavatophorus* only formed raw mass spectra on SAB agar. These results, along with other studies [49], underline the importance of appropriate media (i.e., low a_w_ media) and incubation time, both for constructing and for using the database.

### Implications and future directions

Xerophilic/xerotolerant *Aspergillus* species were detected across multiple environments and sample types though they constituted only a small fraction of all fungi. For future studies, we recommend incorporating low activity media (e.g., MY50) into both initial plating and broth cultivation protocols to obtain more knowledge on the presence of xerophilic/xerotolerant fungi in indoor environments. While this may not be necessary for routine surveys, it could be warranted in cases where environmental conditions, such as dry, climate-controlled buildings, suggest a potential niche for xerophilic species. The data from the warehouse, along with prior reports from museum settings [26,50], indicates that xerophilic fungi may persist even under stringent environmental controls. As such, targeted methods may be crucial for accurate assessment of fungal exposure in vulnerable settings.

## Conclusion

It was possible to construct a supplementary MALDI-TOF MS database successfully which complemented the Bruker database, with the combined databases identifying additional xerophilic/xerotolerant *Aspergillus* species in different environmental samples, previously going undetected, and therefore provided a more in-depth characterisation of various environments. The supplementary database has the strength that spectra are obtained from fungi grown in different broth media.

For future constructions of MSPs of xerophilic/xerotolerant *Aspergillus* species and the subsequent use of the MSPs for species identification we recommend using broth and agar media agar with low water activity and using an extended incubation time of 3 to 5 days in broth media. For *A. halophilicus* an even longer incubation time and double the matrix are needed. Future studies expanding the MALDI-TOF MS database by including more strains of each species may improve the quality of the database and clear observed discrepancies.

## Data Availability

The datasets generated during and/or analysed during the current study are available from the corresponding author on reasonable request.
